# P-cadherin mutations are associated with high basal Wnt activity and stemness in canine mammary tumor cell lines

**DOI:** 10.18632/oncotarget.26873

**Published:** 2019-04-26

**Authors:** Elpetra Timmermans-Sprang, Rob Collin, Arjen Henkes, Meike Philipsen, Jan A. Mol

**Affiliations:** ^1^Department of Clinical Sciences of Companion Animals, Faculty of Veterinary Medicine, Utrecht University, Utrecht, The Netherlands; ^2^Department of Human Genetics, Radboud University Medical Center, Nijmegen, The Netherlands; ^3^Donders Institute for Brain, Cognition and Behaviour, Radboud University Medical Center, Nijmegen, The Netherlands

**Keywords:** cancer stem cell, canine mammary tumor exome-sequencing, CDH3, Wnt, cSRC

## Abstract

**Purpose:** To find underlying mutations causing highly-activated Wnt activity in mammary tumor cell lines associated with rounded morphology indicative of stemness/EMT.

**Methods:** Stemness of high Wnt cell lines was confirmed using qPCR on selected genes and microRNA profiling, followed by whole-exome sequencing of 3 high Wnt canine mammary tumor cell lines and 5 low/absent Wnt cell lines. Candidate genes were identified and their involvement in Wnt activity investigated using siRNA silencing.

**Results:** The high Wnt cell lines had morphological and gene expression characteristics reminiscent of stemness. All individual cell lines had about 4000 mutations in the exome in comparison to the reference canine genome. The three high basal Wnt cell lines had 167 unique exome mutations. Seven of these mutations resulted in a SIFT score <0.2 of proteins related to Wnt signaling. However, gene silencing did not change the Wnt pathway activation. Renewed analysis with respect to putative relations to Wnt signaling revealed that P-cadherin (CDH3) had three mutations in the coding region of the extracellular domain and was associated with high Wnt signaling. Silencing by siRNA not only in lowered Wnt activity, but also decreased levels of phosphorylated cSRC and sP-cad, and changed cell morphology towards spindle cell appearance.

**Conclusion:** It is concluded that expression of mutated CDH3 is associated with activation of cSRC, stabilization of ß-catenin and a rounded morphology related to a stemness/EMT phenotype. A decreased Wnt activity can be found also by cSRC inhibition, but CDH3 silencing has an additional effect on morphology indicating reversal of EMT.

## INTRODUCTION

Breast cancer is one of the most frequently occurring cancers in the world and, with an estimated 1.7 million new cases diagnosed worldwide each year, it remains a global health challenge. Although intensive screening and improved treatment options have increased the 5 year survival rate, still roughly 30% of woman with breast cancer eventually die of their disease due to metastases resistant to therapy [1, 2]. Not only metastases may become resistant to therapy, also luminal, steroid hormone receptor positive cells can lose their sensitivity towards endocrine treatment and form metastases [3, 4]. Therefore, gaining insight into the development of metastasis and therapy resistance is crucial. Cancer stem cells (CSCs) are thought to play an essential role in enhanced self-renewal ability, therapeutic resistance and metastasis [5, 6]. These CSCs may arise from a mutation in a healthy stem cell and lost the ability to regulate its own cell division [6], resulting in CSC overpopulation which drives tumor growth with an intratumor heterogeneity. CSCs can escape and form metastases [7]. Targeting the CSC may be essential in cancer therapy [8–10]. However, CSCs are resistant to chemo- and radiotherapy and therefore new targets should be developed [11]. One of the major signaling pathways for CSCs is the canonical wingless-type MMTV integration site family (Wnt) pathway [5, 6, 12]. Wnt promotes the outgrowth of metastatic lesions and CSCs, breast CSCs have an active Wnt signaling and more than half of the breast cancers have an activated canonical Wnt signaling which is associated with a lower overall survival [13–16]. In both human and canine mammary cancer the enhanced canonical Wnt activity can not be explained by mutations that cause Wnt activation in for instance colon cancer [15, 17].

During recent years, spontaneous mammary cancer in dogs has shown great value for analysis and developing treatment methods for EMT involved signaling mechanisms [18]. The incidence of mammary carcinomas in dogs is high and the pathogenesis is, as in humans, clearly hormone dependent [19]. In recent years we investigated the role of Wnt signaling in canine mammary cancer and found high basal, ligand-independent Wnt activity in a subset of canine mammary tumor cell lines [20]. Classical gene mutations known to activate the Wnt pathway were not found [20]. The high active Wnt cell lines had also a more rounded morphology in contrast to the spindle cell like features of the other cell lines and were further characterized by enhanced mRNA expression of LEF1, HER2/3, and CDH1 and the loss of PTEN mRNA expression. Inhibition of the PI3K pathway by mTor inhibitors, or silencing of HER1-3 even further stimulated basal Wnt activity that appeared to be sensitive to cSRC inhibitors [21–23].

Metastases can be formed from epithelial cells that undergo epithelial-mesenchymal transition (EMT) resulting in cells with phenotypically stromal cell characteristics. During EMT cells detach from neighboring cells and the basal membrane, obtain a rounded morphology and acquire migratory capacity. This process can also occur in cancer cells at the invasive front of the tumors [24]. Among others, changes in expression of cell adhesion molecules such as cadherins or integrins play an important role in EMT. When cells are in transition between epithelial and mesenchymal with intermediate phenotypes specific detection of markers is impossible [25]. Micro-RNAs (miR) play an essential role in tumorigenesis. In breast cancer (BC) cell lines, certain sets of miRs are related to EMT and/or stemness [26]. Major drivers of EMT are upregulation of the Wnt pathway and loss of E-cadherin (CDH1). Both factors are significantly correlated with poor outcome in breast cancer [27]. CDH1 is a key cell-to-cell adhesion molecule; loss of CDH1 expression in carcinomas will dissemble epithelial cell sheets. Increased CDH1 expression was therefore thought to be an antagonist of invasion and metastasis [24]. However, basal-type breast cancer cell lines can undergo EMT without losing their CDH1 expression [28]. Both E-cadherin (CDH1) and P-cadherin (CDH3) are members of the super family of cadherins and major components of cell-cell adhesive junctions. The main region responsible for cadherin’s adhesive properties is extracellular domain 1 (EC1). EC1 forms a zipper-like structure between cells [29]. In addition to their adhesive function both cadherin’s have a function in the biology of stem cells [30, 31]. They activate downstream FAK, rous sarcoma proto oncogene (cSRC) and AKT kinases, EGFR and Wnt signaling [28, 30, 32]. Cadherins bind β-catenin, an essential component of Wnt signaling, that once free and stabilized in the cytosol migrates to the nucleus to stimulate Wnt-target genes.

As the question remained unanswered why our cell lines show this highly basal activated Wnt activity and a rounded morphology despite high E-cadherin expression, we decided to perform a whole exome sequencing analysis to find mutations unique for the high basal Wnt active canine mammary tumor (CMT) cell lines and to relate candidate genes to upregulated Wnt activity. These candidate genes were further investigated using mRNA silencing experiments. At the end we found that the candidate gene P-cadherin, with three extracellular domain missense mutations, was causing both Wnt activity and responsible for the morphology changes. Silencing of these P-cadherin mutations in EC1 showed that the Wnt activity is reduced and rounded cells got their spindle cell morphology back. The mutations may result in a possible enhanced sensitivity for proteolytic cleavage of the extracellular domain of P-cadherin and so stimulate Wnt signaling and morphological changes.

## RESULTS

### Markers of stemness in canine mammary tumor cell lines

**Figure 1:**
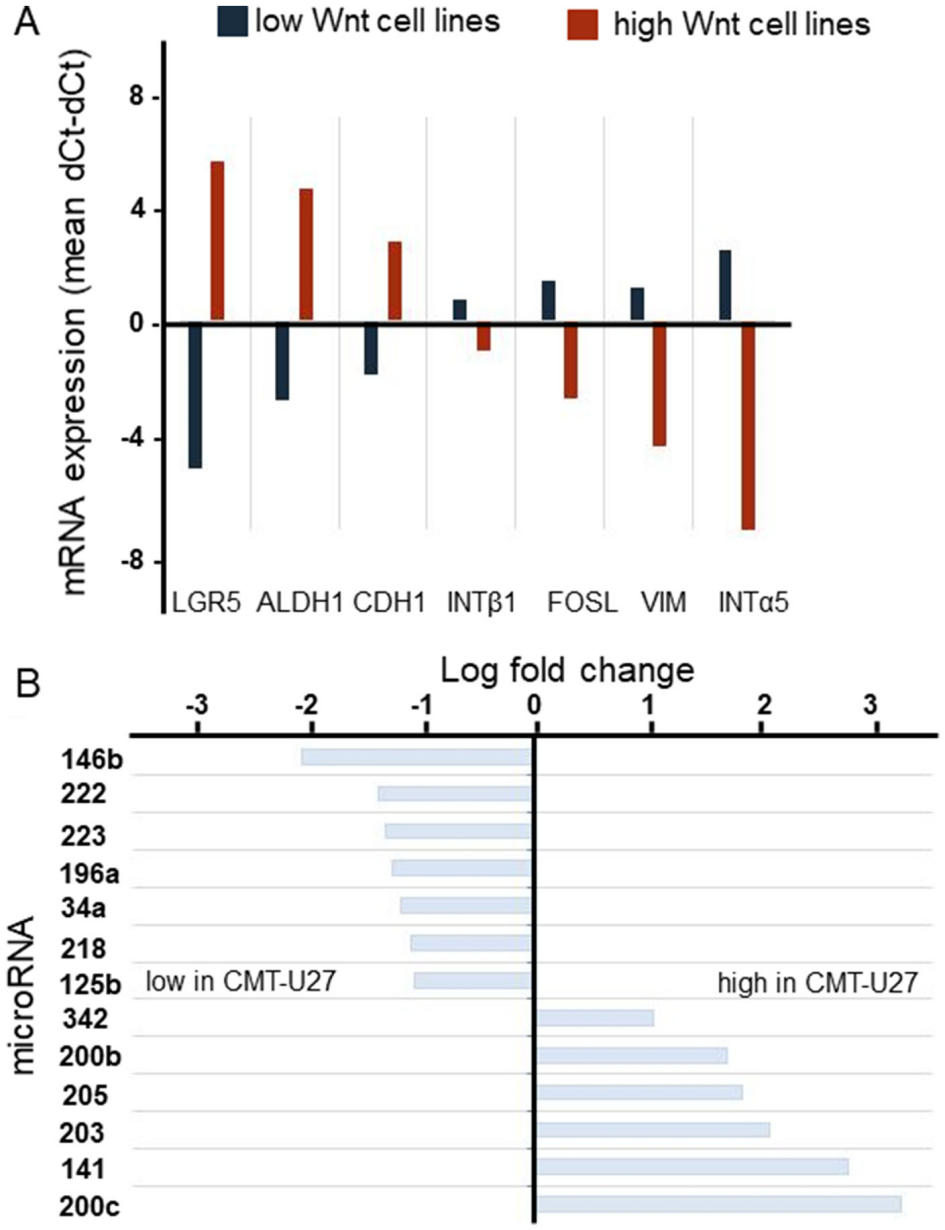
The genotyping of canine mammary tumor cell lines. mRNA expression of stem cell markers was analyzed in 8 canine mammary tumor cell lines. For each cell line 2 different passages were used. The average of the low (blue) active Wnt cell lines (CHMm, CNMm, CIPp, CIPm and P114) were presented against the average of the high active Wnt cell lines (red) (CMT1, CMT-U27 and CMT9) (**A**). miR levels of HER2/3 overexpressing, PTEN-negative CMT-U27 cells with highly activated basal Wnt activity canine mammary tumor cell line CMT-U27 were compared to those CIPm cells with low Wnt activity. With the miFinder array 90 different miRs were verified. MicroRNAs with a fold change difference >2 times were presented. In the left panel the microRNAs with a lower miR expression level in the CMT-U27 cells are shown (**B**).

In contrast with the spindle cell morphology of the majority of canine mammary tumor cell lines, the cell lines with high basal, ligand-independent, Wnt activity were characterized by a rounded morphology reminiscent of stemness or EMT. To further characterize these cell lines the relative expression of a variety of additional markers was measured by qPCR (Figure 1A). In addition, a miRNA expression profile comparison was made from a low and high Wnt signaling cell line. The (cancer) stem cell markers *ALDH1A1* and *LGR5* were expressed at significantly higher in the rounded Wnt active cells. Expression of the epithelial marker *CDH1* was higher, whereas the expression of mesenchymal markers *INTα*5, *INTβ*1 and *VIM* was significantly lower in the cells with rounded morphology. *FOSL1* expression, involved in migration and invasion was lower. The most striking differences in the miR expression profile related to Wnt signaling and stemness were the low expression of miR-34a, -146b and -196a in the high active Wnt canine mammary tumor cell line CMT-U27, and the high expression of miR-200b, -205 and 200c compared to the control cell line CIPm (Figure 1B). As will be discussed, these findings are in line with the enhanced Wnt activity and low *INTα*5 expression but atypical for stemness and EMT.

### Exome sequencing

**Figure 2:**
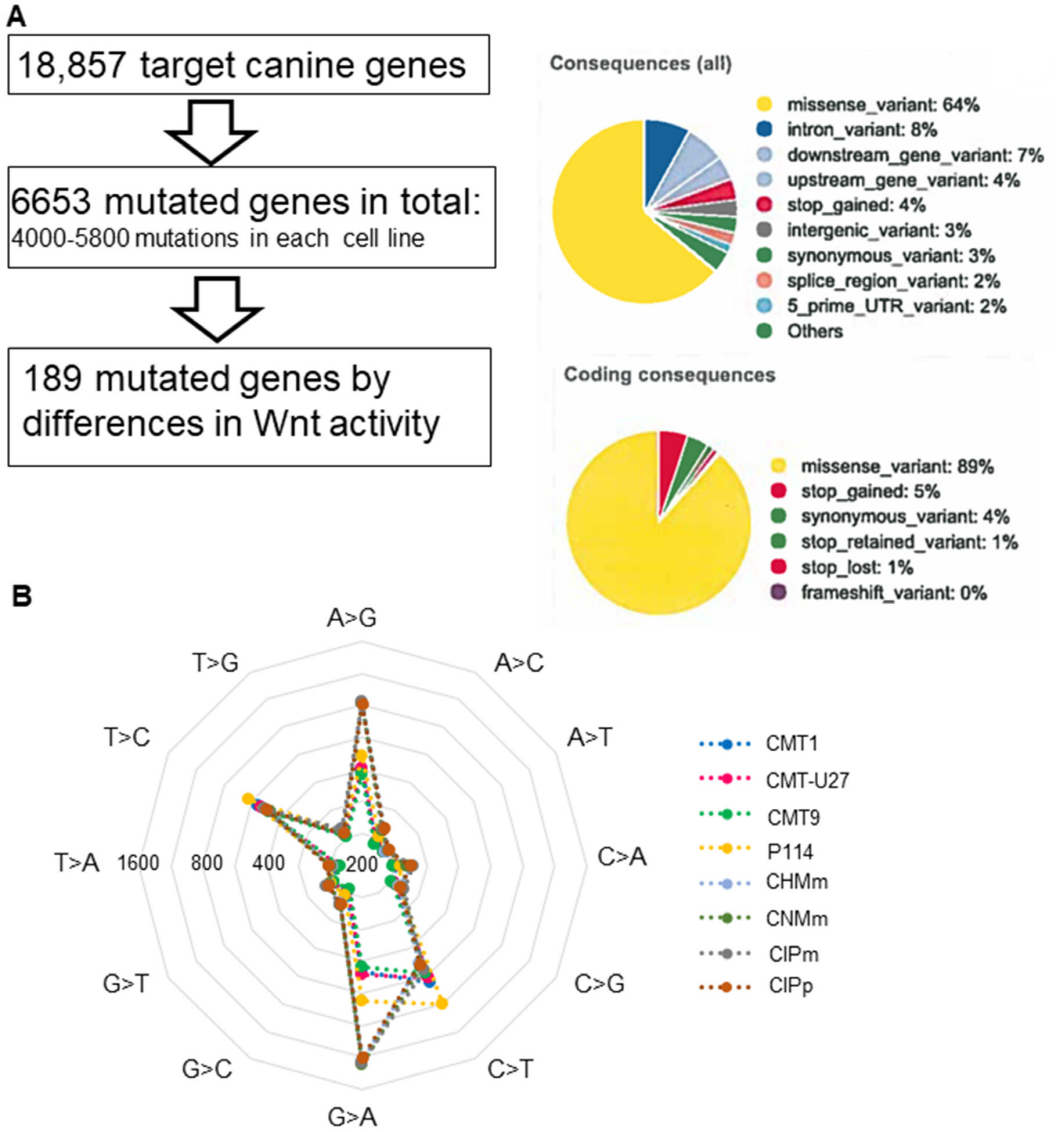
The exome sequencing in 8 canine mammary tumor cell lines. Results of canine mammary tumor cell lines exome sequencing, an analysis pipeline and mutation overview. Most were missense mutations in the coding region (**A**) and most mutations are common in all cell lines and were from G>A (T>C) and A>G (C>T) (**B**).

**Figure 3:**
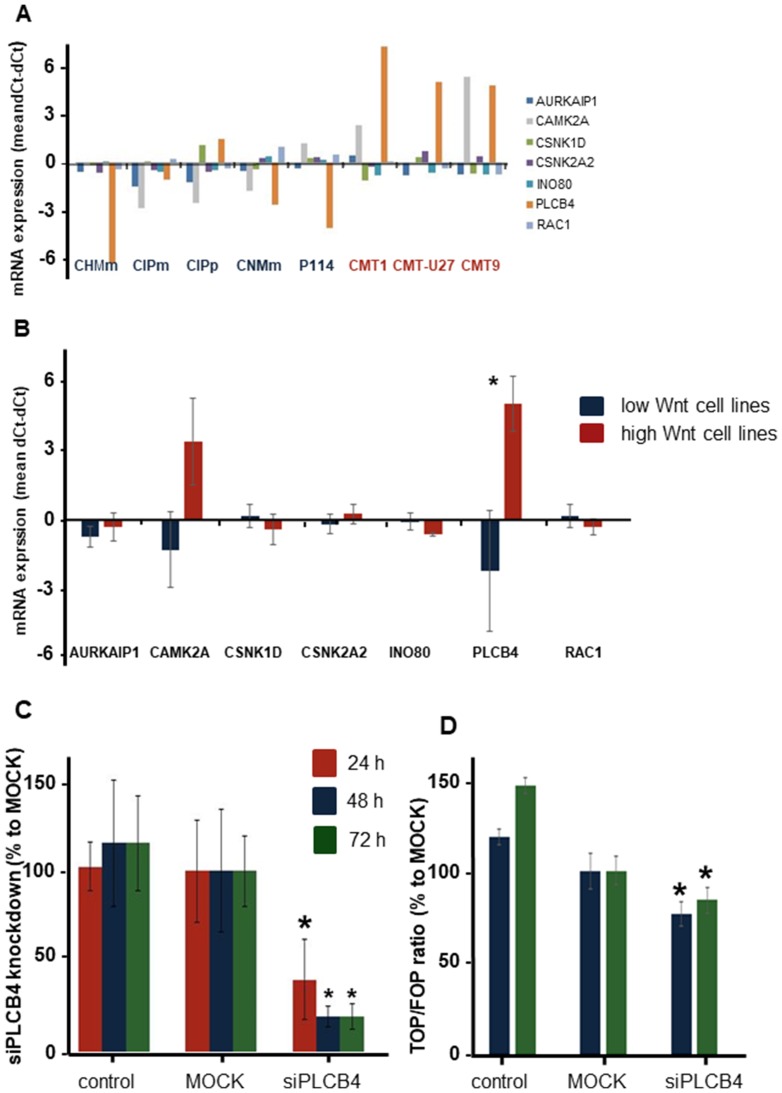
The exon mutation or splice side mutation in 7 genes in relation to the Wnt pathway. These genes were further investigated on differences in mRNA expression (**A**). PLCB4 gene was the only significantly different gene between the low and high Wnt active cell lines (**B**). Knockdown of PLCB4 with a siRNA gave a 60 to 80% reduction in mRNA expression (**C**). This decrease in PLCB4 mRNA resulted in 23 and 16% significant reduction in the TCF reporter activity (**D**).

The high basal Wnt activity of 3 mammary cell lines can only be explained by mutations in the Wnt-signaling pathway. As no mutations were found in classical mutated genes of the Wnt pathway, i.e. APC, ß-catenin, or GSK3ß, we decided to sequence the complete exome of eight canine mammary tumor cell lines; three cell lines with activated basal Wnt signaling and a rounded morphology and five cell lines with a low Wnt activity and a spindle cell morphology. We compared the exome sequencing results with the published canine CamFam3.1 genome and found between 4000–5800 mutations in the individual cell lines. Within the coding regions 89% of the mutations were missense variants (Figure 2A). In the low Wnt signaling cell lines more missense mutations were found (on average 5542 vs 4288, *P* < 0.05). Most of the mutations were G>A or A>G (Figure 2B); however these were not different among the various cell lines and not associated with high basal Wnt activity. Mutations unique for the high active Wnt cell lines. A number of genes were selected for further analysis. *INO80*, *AURKAIP1* and *PLCB4,* had mutations that were most detrimental for protein function as shown by a “sorting tolerant from intolerant” (SIFT) score ≤0.1. *CAMK2A*, *CSNK2A2* and *RAC1* had a splice donor (SD) or splice acceptor (SA) mutation. *CSNK1D* was selected although it was only mutated in CMT-U27 and CMT9 (Supplementary Table 1). CDH3 had more mutations in both the high and low active Wnt cell lines but also three specific mutations in the high active Wnt cell lines in the extracellular domain (EC). The mRNA expression levels of these selected target genes were measured. Differences in expression levels were found among the cell lines (Figure 3A) of which only PLCB4 expression was significantly higher in the high activated Wnt cell lines (Figure 3B). Silencing of *PLCB4* mRNA using specific siRNA resulted in a knockdown of 80% with only a 20% decrease of the basal Wnt activity as shown by the measurement of luciferase activity of a TCF-sensitive luciferase reporter construct (TOPflash) relative to the same construct with inactivation mutations in the TCF binding site (FOPflash) (Figure 3C and 3D). All these mutated tested genes with a low SIFT score, splice donor or acceptor mutations did not reduce the highly activated Wnt signaling to moderate reporter activity comparable to human mammary cell lines [33, 34]. Therefore we searched in the exome sequence data for other putative Wnt related genes irrespective of the SIFT score to find an association between the activated Wnt pathway and the morphology changes in the CMT cell lines.

### CDH3

**Figure 4:**
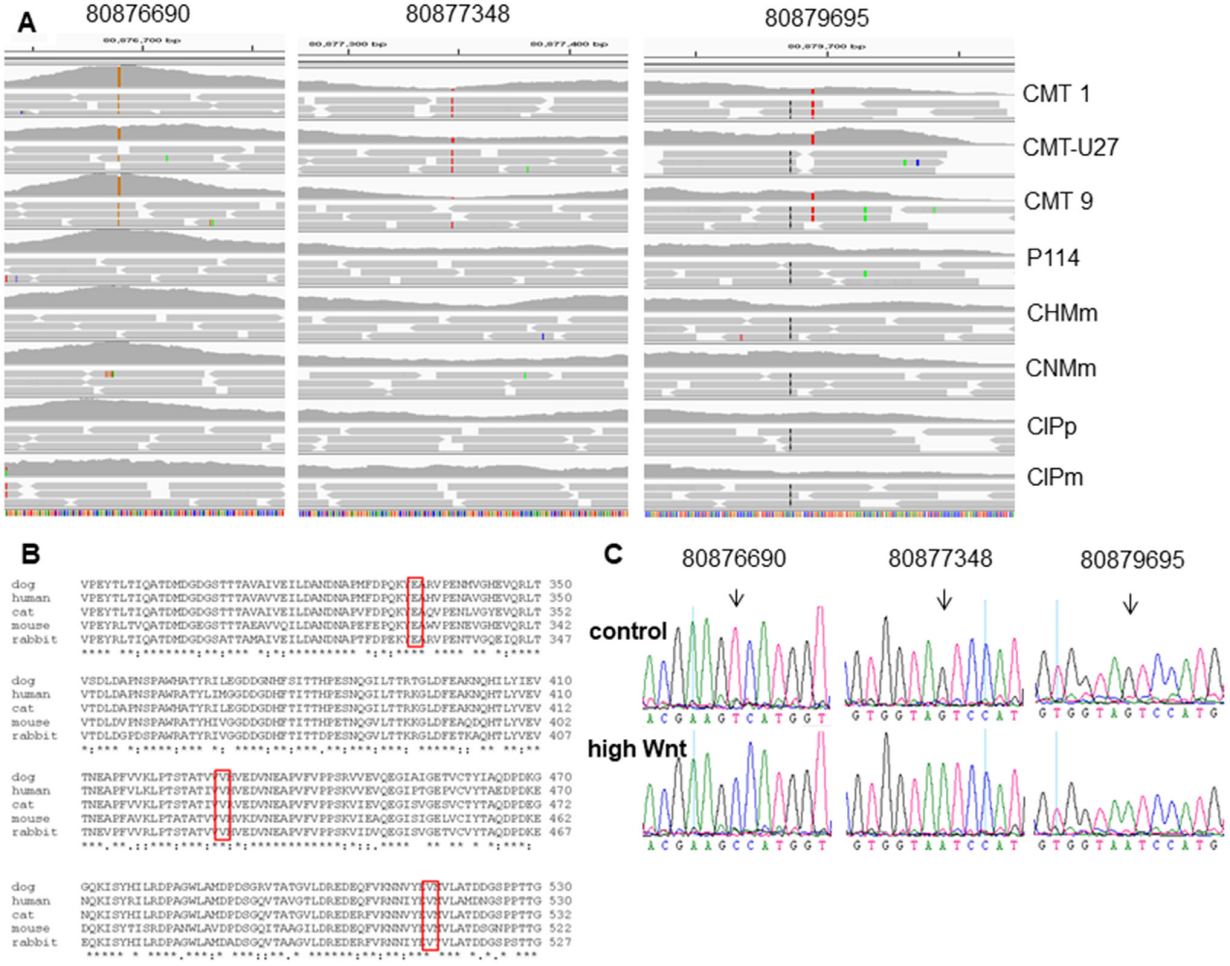
The CDH3 mutations in chromosome 5 on locations: 80876690, 80877348 and 80879695 (min-strand). (**A**) Schematic representation, visualized with the Integrative Genomics Viewer (IGV version 2.3.59) browser against CanFam 3.1 dataset, of the mapped exome sequencing around chromosome 5 of 8 canine mammary tumor cell lines (80876650-80879700 min strand). (**B**) Clustal O protein alignment of Canis lupis familiaris (XM_005620789) with 829 amino acids (AA), Homo sapiens (NM_001793), Mus musculus (NM_001037809), Oryctolagus cuniculus (XM_008257527), Felis catus (XM_019819960). AA 334 from the dog (80879695) mutated from Glutamic acid, GLU E (GA (A, G)) to Lysine, Lys K (AA(A, G)). AA 429 (80877348) mutated from Valine, Val V (GTx) to Isoleucine Ile I (AT (TCA)). And AA 516 (80876690) mutated from Val (GTx) to Alanine Ala A (GCx). (**C**) Sanger sequencing confirms the mutations identified by exome sequencing. Chromatograms of a control cell line and a basal highly activated Wnt canine tumor cell line. Mutations are depicted for each variant (black arrow).

**Figure 5:**
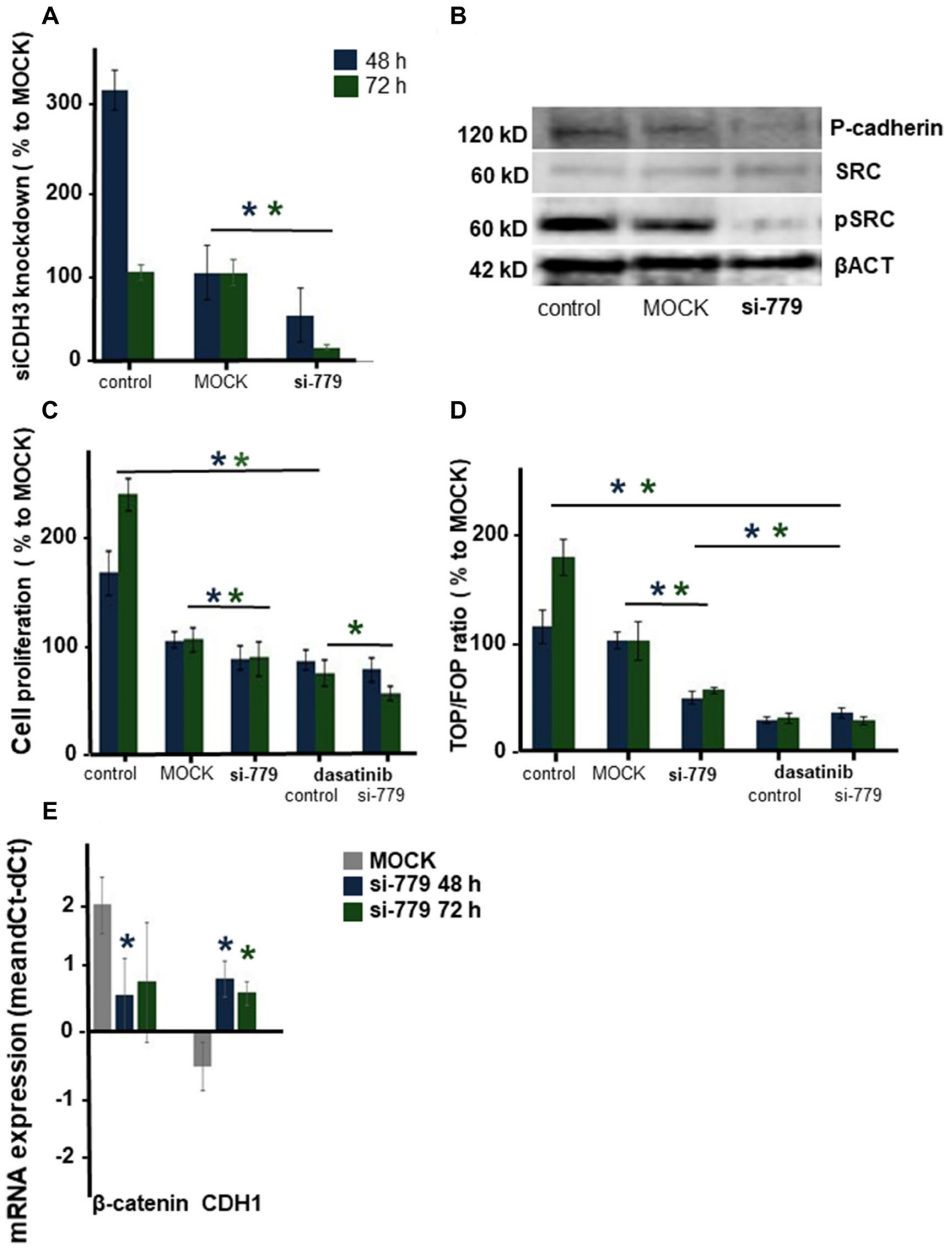
The effect of silencing CDH3 mRNA in CMT-U27 canine mammary tumor cells. mRNA was silenced on 779 in EC1. CDH3 expression was analyzed in CMT-U27 cells that were nontransfected (control) or transfected with the MOCK control (MOCK) orCDH3-779 siRNA. mRNA expression was analyzed 48 and 72 h post-transfection. Tested for equal distribution and significance with a *t*-test in Excel, 3 independent experiments with *n* = 4, ^*^P < 0.05 (**A**). Protein expression level for CDH3 (Ab human P-cadherin AA 72-259), cSRC, pSRC and βACT 72 h after silencing (**B**). Cell proliferation in (**C**) was represented as a percentage of the MOCK. CMT-U27 cells were also treated with dasatinib (20 μM). Statistically analysis was done on one representative experiment, out of three experiments (*n* = 8). Normal distribution was tested and *t*-test performed in Excel, ^*^P < 0.05. Wnt activity was analyzed with the TCF reporter TOP or FOP transfection assay (**D**). After 48 and 72 h the TOP/FOP ratio was analysed with a Dual-luciferase assay and corrected for the Renilla efficiency. The mean ratio was expressed of one representative experiment (*n* = 4), this experiment was repeated three times and statistically tested for normal distribution and significance with a *t*-test in Excel, ^*^P < 0.05. Target gene mRNA expression of β-catenin and CDH1 was showed in (**E**) *n* = 6 statistically tested for normal distribution and significance with a *t*-test in Excel, ^*^P < 0.05.

**Figure 6:**
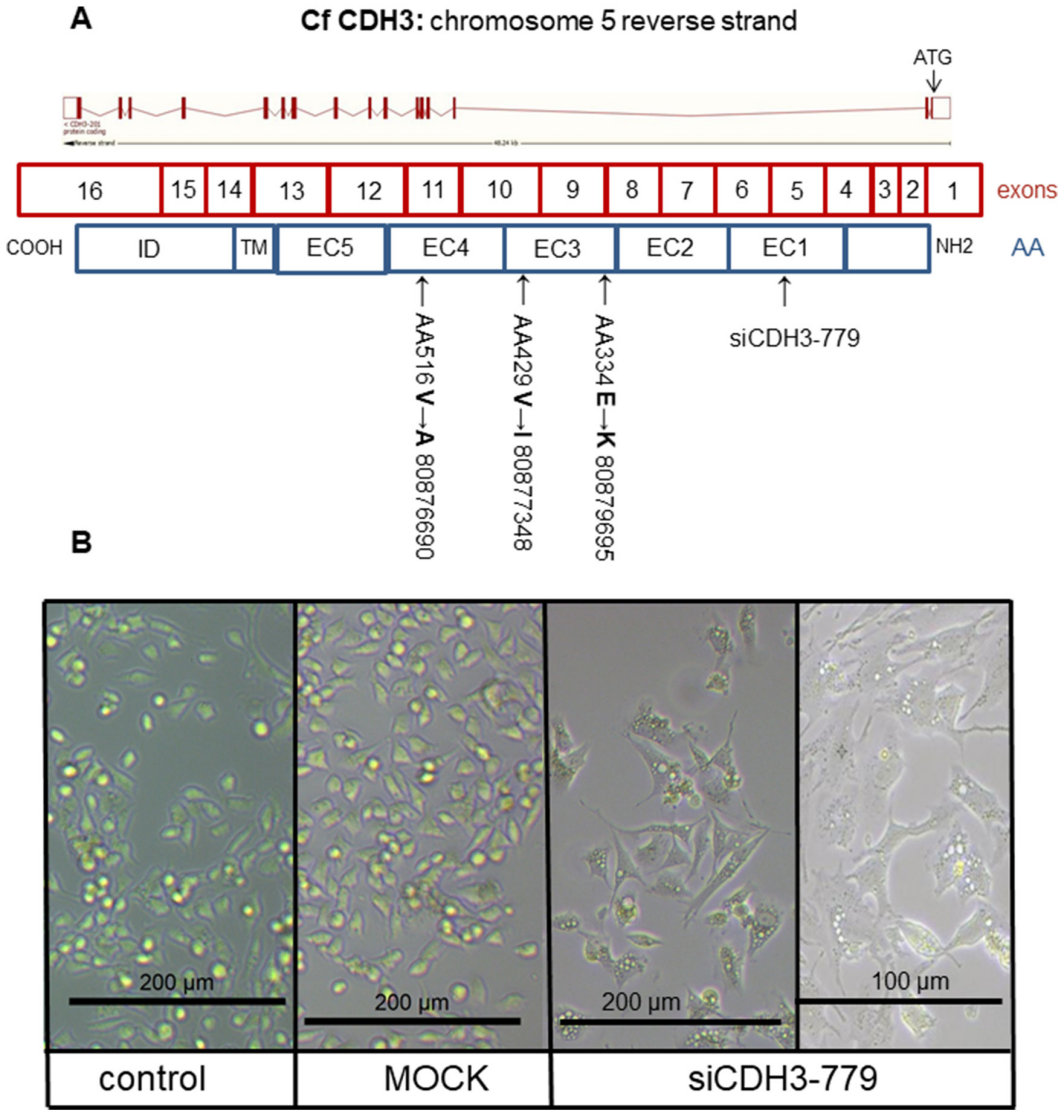
The CDH3 structure and morphology. Schematic representation of canine CDH3 DNA organization on chromosome 5: 80,864,778-80,913,018 reverse strand with 16 exons (4196 bp) resulted in 829 aa. Most of the protein was extracellular (650 aa), the remaining was transmembrane (27 aa) and cytoplasmic (152 aa). Silencing location was in extracellular domain (EC) 1 (siCDH3-779). Two missense mutations were in EC3 (80,879,695 and 80,877,348), one missense mutation in EC 4 (80,876,690) (**A**). CMT-U27 morphology changes in (**B**). The non-transfected control and MOCK showed rounded cells with bright field microscopy. Cell morphology of CDH3 silenced in EC 1 (siCDH3-779) changed to a spindle cell structure 72 h after silencing (all at 200 μm and siCDH3-779 also at 100 μm).

Next, we investigated the role of the ß-catenin binding cell-to-cell adhesion protein P-cadherin encoded by the *CDH3* gene. Screening the exome sequencing data showed 3 mutations in *CDH3* with high SIFT scores, meaning that the impact of these individual missense mutations on protein activity is predicted to be low. Nevertheless, the influence of these three single amino acid substitutions was further investigated. The Integrative Genomics Viewer (IGV) showed for all three missense mutations more than two reads (Figure 4A). On canine chromosome 5, on nucleotide (nt) 80876690 of the minus strand a T>C transition resulted in an amino acid change from Val to Ala (SIFT score 1), at nt-80877348 a G>A transition resulting in a Val > Ile change (SIFT score 0.36) and at nt-8079695 also a G>A base transition resulting in a Glu > Lys change (SIFT score 0.34) (Figure 4C). All three missense mutations were only found in the high active Wnt cell lines CMT1, CMT-U27 and CMT9, changing amino acids that are well-conserved in human, cat, mouse and rabbit CDH3 (Figure 4B). The mutations were found in exon 9, 10 and 11 encoding the EC3 and EC4 of *CDH3* (Figure 6A). To test the effect of silencing CDH3 mRNA, a siRNA for *CDH3*, on EC1 (nt-779) of the published cDNA was tested on mRNA level, protein expression, cell proliferation and Wnt activity and on CDH3 target genes in CMT-U27 cells. Silencing *CDH3* had a significant knock down on mRNA of 50% at 48 h and 85% at 72 h normalized to the MOCK (Figure 5A). The differences between the control and MOCK cells at 48 h, were caused by the lipofectamine/siRNA treatment that was necessary for the silencing of the non targeting pool in the MOCK and with si*CDH3*, resulted in a recovery of the cells after 72 h (Figure 5C). Silencing *CDH3* on EC1 (siCDH3-779) resulted in decreased P-cadherin protein expression (Figure 5B), a small but significant decrease in cell proliferation (Figure 5C) and inhibition of Wnt activity 52% and 45% after 48 h and 72 h, respectively (Figure 5D). It was previously shown that the Wnt activity was also sensitive to cSRC inhibition by dasatinib. We therefore tested the effect of *CDH3* silencing on SRC and phosphor-SRC expression and found a major inhibition of pSRC (Figure 5B), an additive effect on inhibition of cell proliferation, but no further decrease of the dasatinib mediated decrease in Wnt activity (Figure 5D, 5E).

**Figure 7:**
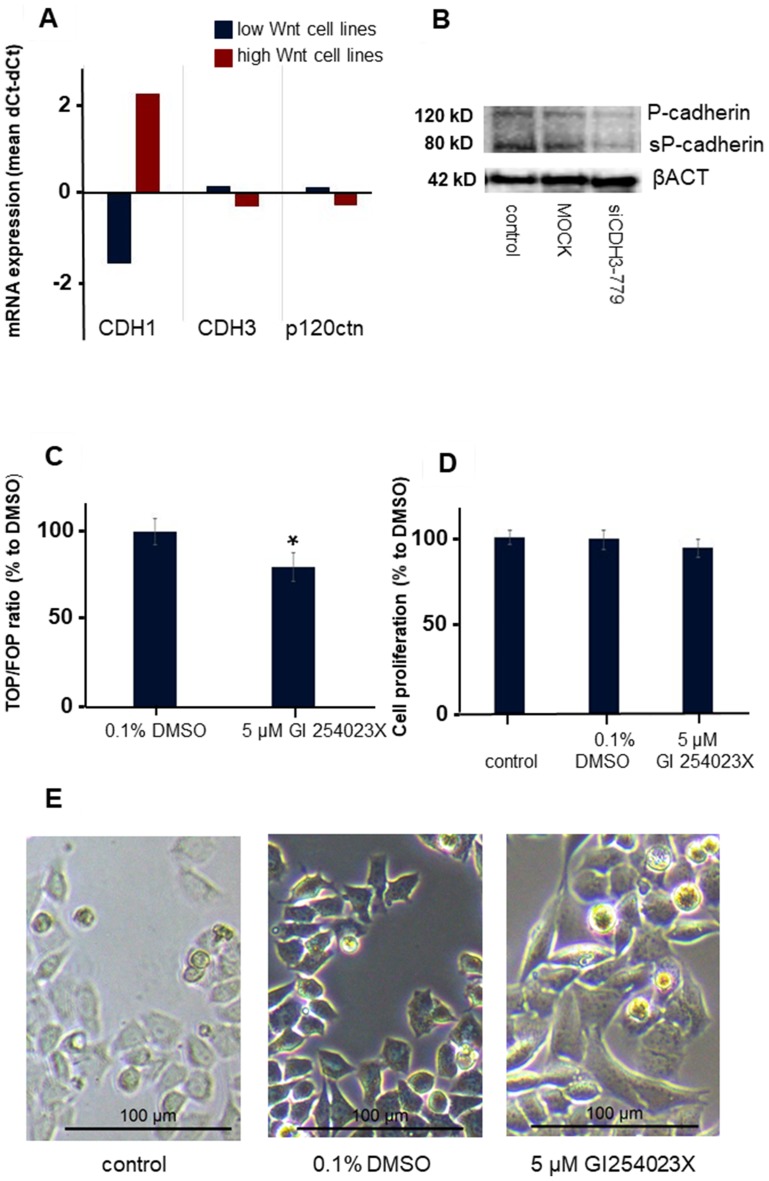
The effect of ADAM10 inhibitor GI 254023X in the CMT-U27 canine mammary tumor cell line. Differences in mRNA expression levels in canine mammry tumor cell lines with low and high Wnt activity (**A**). Western blot analysis of P-cadherin, sP-cad and βACT after siRNA treatment for 72 h with siCDH3-779 in CMT-U27 (**B**). Wnt activity (TOP/FOP ratio) was analysed after 48 h treatment of CMT-U27 cells with a selective ADAM10 inhibitor *n* = 6 and significance was tested with a *t*-test in excel ^*^P < 0.01 compared to the DMSO control (**C**). The effect on cell proliferation of this inhibitor on CMT-U27 after 48 h (**D**) *n* = 8 and morphology changes in (**E**). The control and DMSO treated cells showed rounded cells with bright field microscopy. Treatment of CDH3 mutated CMT-U27 cells with an ADAM10 inhibitor changed the morphology to a spindle cell structure in 48 h.

Most remarkable was the change in morphology of the normally rounded CMT-U27 cells. Within 72 h after silencing *CDH3* in EC1, just before the mutations in EC3 and EC4, cells became more stretched and regained a spindle cell shape (Figure 6B). No differences in mRNA expression of *CDH3* and *p120ctn* was observed between the high and low Wnt activated cell lines (Figure 7A). Silencing *CDH3* in EC1 in CMT-U27 cells with high Wnt avtivity, reduced also the amount of sP-cad protein (Figure 7B). Whether this was caused by enhanced proteolytic cleavage was investigated with an ADAM10 inhibitor, GI 254023X. With this inhibitor the Wnt signaling was partially reduced to 80% (Figure 7C) without effecting the CMT-U27 cell proliferation (Figure 7D) and cells became more stretched and regained a spindle cell shape in 48 h (Figure 7E).

## DISCUSSION

In this study, we showed that 3 missense mutations in the extracellular domain of CDH3 are the basis of a morphological change from spindle cell to rounded cells and a high basal, ligand-independent, Wnt signaling activity. The high activated Wnt cells are not only characterized by a rounded cell morphology and high Wnt activity, but also by relative high expression of the mRNAs of *CDH1*, *LEF1*, *HER2* and *HER3* and loss of *PTEN* expression all of them may contribute to an environment contributing to enhanced Wnt activity [20, 21, 23]. Additional markers and siRNA profiling showed high expression of *ALDH* and *LGR5* in agreement with the stem cell characteristics of breast cancer cells [10, 35]. ALDH1 affects proliferation and early differentiation of breast stem cells [36], and LGR5 serves as a functional factor in CSCs by activating the Wnt pathway [35, 37, 38]. However, the high expression of epithelial marker *CDH1* and low expression of mesenchymal markers *VIM* and *ITGα5* is atypical for classical EMT [20, 25, 28]. Additionally, the high expression of miR-200c and miR-203, which are potent inhibitors of EMT [39], questions the transition towards a mesenchymal phenotype despite the morphology of these cells. The low expression of *ITGα5* can be attributed to the high miR-205 expression [40]. The low expression of miR-146b, miR-222, miR-34a and miR-125b, all negative regulators of Wnt signaling [6, 41, 42], are in agreement with a CSC phenotype. The high miR-141 and miR-200c expression and low miR-196a was also found in HER2 overexpressing human breast cancer cell lines [26]. It is therefore concluded that our high active Wnt canine mammary tumor cells are best described as HER2 positive cells with an enhanced CSC phenotype.

From the exome sequencing data we found 189 genes to be uniquely mutated in the three high activated Wnt cell lines. From these genes a selection was made on genes known to have a known relation to the Wnt pathway: *INO80*, *AURKAIP1*, *CSNK1D*, *CSNK2A2*, *CAMK2A*, *RAC1, CDH3* and *PLCB4*. All these genes are involved in the canonical, non-canonical or planar cell polarity (PCP) Wnt pathway [43–48]. As a first approach mRNA expression analysis was performed revealing enhanced expression of *PLCB4* mRNA in the high activated Wnt cell lines. *PLCB4* encodes the β4 isoform of phosphoinositide-specific phospholipase C (PLC) isoenzymes, a member of a superfamily that regulates the metabolism of inositol lipids. Although PLCB4 is a key hub gene in Wnt signaling in MCF7 cells [49], silencing *PLCB4* mRNA in CMT-U27 only inhibited Wnt activity measured with TOP/FOP reporter ratio by about 20%.

As the morphology change of these cells was obvious and Wnt-signaling pathways can cross-talk with cell adhesion and morphogenesis by cadherins we investigated next the role of cadherins in our cells [32]. Silencing CDH3 with siCDH3-779, targeting the *CDH3* mRNA in the area encoding the EC1 domain, was effective in downregulation of P-cadherin protein content, Wnt activity and inhibition of cell proliferation. In addition it changed themorphology from rounded to spindle cell, inhibited the phosphorylation of cSRC and reduced the amount of sP-cad.

In BC cells EMT is defined by morphological changes in combination with a loss of E-cadherin. Reconstruction of WT E-cadherin in MDA-MB-231 cells that had undergone EMT with a loss of E-cadherin did, however, not change the cell morphology suggesting that loss of E-cadherin is not necessary for EMT [28]. The presence of P-cadherin in an E-cadherin positive cellular background, as is also the case in our cells [20], may nevertheless stimulate an invasive phenotype [50, 51]. The enhanced expression of *CDH1* mRNA after silencing of *CDH3* may have contributed to the return of a spindle cell morphology, as CDH1 is also a key cell-to-cell adhesion molecule, like CDH3 [24]. Moreover, the effects of CDH3 are dependent on the expression of CDH1 which is generally low to absent in our canine mammary tumor cell lines with low basal Wnt activity.

E-cadherin and P-cadherin are members of the same superfamily of cadherins and are the major components of cell-cell adhesive junctions. The EC1 domain is most responsible for the adhesive junctions by making a zipper-like structure between the cells with a preference for homotypic adhesion. The intracellular domain of both cadherins can form a complex with ß-catenin and cSRC [32] resulting in lower free cytoplasmic β-catenin and consequently lower Wnt activity [29, 50]. In contrast to the suppressor role of *CDH1*, *CDH3* may act as an oncogene and stimulates a pro-invasive character of tumor cells in the presence of *CDH1* resulting in a poor prognosis [50]. This is also shown for colon cancer cells where knockdown of *CDH3* also resulted in reduced β-catenin expression and upregulated *CDH1*, called the cadherin switch [30, 52]. CDH3 is overexpressed in highly malignant breast tumors [53], and is an independent prognostic factor in lymph node malignancy [54].

P-cadherin is also associated with undifferentiated normal epithelial tissue and poorly differentiated breast carcinomas, breast stem cell markers and features linked to the aggressive behavior of basal like breast cancers [30, 55]. This aggressiveness comes from sP-cad that is produced by membrane metalloproteolytic enzymes (MMPs) and is responsible for the invasive capacity of breast cancer cells [56]. This MMPs affected P-cadherin more than E-cadherin [56]. Whereas reconstruction of wild-type CDH1 in human breast cancer cell line MDA-MB-231 didn’t revert the morphology of the human cells [28], treating this cells with the ADAM10 specific inhibitor, GI 254023X showed a reduced migration without effecting cell numbers [57]. This is in agreement what we observed in the CMT-U27 cells with high Wnt activity and three missense mutations in *CDH3*. The tested ADAM10 inhibitor partially reduces the upregulated Wnt activity in the CMT-U27 cells with high Wnt activity and regained the morphology to a more spindle cell. It is reasonable that the three missense mutations in *CDH3* increases the sensitivity to cleavage of P-cadherin resulted in the dissociation of the p120ctn, β-catenin and SRC complex [32, 56–58].

Silencing of *CDH3* on EC1 resulted in a decrease of P-cadherin, sP-cad and phosphorylated cSRC. Treatment of these cells with the cSRC inhibitor dasatinib or with the ADAM10 inhibitor GI 254023X, inhibited the Wnt activity, migration and metastasis [21, 23, 57], suggesting that the enhanced Wnt activity by mutated P-cadherin is mediated by activation of cSRC and enhanced sensitivity for proteolytic cleavage, resulting in stabilizing cytoplasmic β-catenin. The changes in cell morphology are Wnt-independent and likely caused by dissociation of cell-cell junctions [59].

Silencing of three specific *CDH3* mutations on EC 3 and EC4 in canine HER2-positive cells with cancer stem cell characteristics reduced the Wnt activity and changed the morphology of the cells from rounded to spindle shaped. Knockdown of this mutated *CDH3* resulted in decreased Wnt signaling, decreased phosphorylation of SRC, decreased P-cadherin and sP-cad protein. As the Wnt activity in the CMT-U27 cells with high Wnt activity is known to be sensitive to cSRC inhibition, it is conceivable that the mutated *CDH3* stimulates the Wnt activity by activation of cSRC by phosphorylation. However, cSRC inhibition does not result in a morphological reversion to a spindle cell morphology. Inhibiting P-cadherin sensitivity for MMP cleavage have a limited down regulation of the highly basal activated Wnt activity but is more responbible for the morphology changes. Therefore, it is concluded that the three missense mutations in extracellular domain of *CDH3* may result in a possible enhanced sensitivity for proteolytic cleavage. Claeved P-cadherin can activate the Wnt signaling and can change the morphology to a more CSC phenotype of the canine mammary tumor cells.

Further investigation is nessecarry to elucidate if there are more proteases involved for the Wnt activation and to find possible new therapeutic active inhibitors for BC patients with enhanced Wnt activity and a lower overall survival.

## MATERIALS AND METHODS

### Cell culture

Canine mammary tumor cell lines used in this study were established from primary tumors diagnosed as carcinoma (CMT-1, CMT-U27, CMT-9, P114, CHMm, CNMm, CIPp) or from a metastasis (CIPm) [60–62]. For detailed studies, the well-described canine cell line CMT-U27 (a generous gift from Prof. Dr. Hellman, SLU, Uppsala, Sweden) were used. The cell lines were cultured in DMEM/F12 (Invitrogen, Bleiswijk, The Netherlands) supplemented with 10% fetal bovine serum (FBS) (Invitrogen), mycoplasma free tested with Mycosensor QPCR assay (Agilent technologies, Santa Clara, CA, USA) and used with a maximum passage number of 6. CMT-U27 was further characterized by [63–65].

### RNA isolation, cDNA synthesis, sequencing and quantitative RT-PCR

From all cell lines, total RNA was isolated from two different passages for target gene screening. Total RNA was isolated and treated with deoxyribonuclease using an RNeasy mini kit (Qiagen, Venlo, The Netherlands) according to the manufacturer’s protocol with a DNase (Qiagen) treatment. cDNA synthesis was performed using an iScript kit (Bio-Rad Laboratories, Veenendaal, The Netherlands) according to manufacturer’s protocol. Specific primer sets were used to amplify the gene product for quantitative RT-PCR and sequencing (Table 1). Quantitative RT-PCR was performed using Bio-Rad CFX detection system with CFX software (version 3.1) (Bio-Rad Laboratories) with SYBR Green fluorophore. Relative target gene expression was normalized to that of the geNorm tested reference genes using the delta Ct method [21, 66]. Relative induction of gene expression was statistically assessed using paired, two tailed Student’s *t* test after normal distribution testing in Microsoft Office Excel (version 2010) or with SPSS using IBM SPSS Statistics version 24 software (SPSS Benelux, Gorinchem, The Netherlands). Sanger sequencing was done with platinum Taq (Invitrogen) amplified PCR products according to manufacturer’s protocol. DNA sequence reactions were performed using BigDye v 3.1 (Applied Biosystems, Foster City, CA) according to manufacturer’s protocol. All amplifications were performed on an ABI 3130XL (Applied Biosystems) and analyzed in Lasergene (DNASTAR version 14). The obtained sequences were compared with DNA sequences in databases using BLASTn (http://blast.ncbi.nlm.gov/Blast.cgi).

**Table 1:** Primers used to assess gene expression and sequence coging regions

OMIM	Symbol	Forward primer	Reverse primer	Ta	Size	Accession
				(°C)	(bp)	number
**Target genes**						
AURKAIP1	AURKAIP1	ACTACCTCCTACCCAGAC	GAACTTGATCTGCTTCCG	58	265	XM_843641,3
ALDH1A2	ALDH1A2	AGGAGTGTTGAACGGGCTAAAAAG	AACGGTGGGCTGGATGAAGAA	66.5	194	NM_001286977
CTNNB1	β-catenin	ATGGGTAGGGCAAATCAGTAAGAGGT	AAGCATCGTATCACAGCAGGTTAC	64	106	XM_014106479
CAMK2A	CAMK2A	TGCCAGCCACTGTATCCA	AAAGTCCGCCAGTTCAC	60.5	139	XM_005619301.1
CDH1	E-cadherin	CAGGAAGCTCTCCACCAGAG	CTGGGAAATGTGAGCACCTC	58	104	NM_001287125.1
CDH3	P-cadherin	GCCCCGCCCTATGACTCCCTATT	GAAGCGGCTGCCCCACTCG	67	137	XM_005620790.2
CSNK1D	CSNK1D	ATGCTCAAATTTGGAGCCAG	GAGGAGAGGTGTTAGCAGTG	66.5	199	XM_005623972.1
CSNK2A2	CSNK2A2	CAACTATGACCAGTTGTTCG	GTTTGTCCAGAAGATCTAGGG	63	200	XM_005617496.1
FOSL1	FOSL1	TCCTCGGGGCCTGTGCTTGAAC	CCGCTGCTGCTGCTGCTGCTG	66.5	154	XM_022405633
INO80	INO80	AACACATACTCGCTGAATGG	CCTCACTGGACTCATCAC	58	132	XM_005638259,1
ITGA5	ITGα5	ATACCCGGATCTGATTGTG	GTTGAGGCAGAAGCTGAG	62	180	XM_005636739
ITGB1	ITGβ1	CAGTTACAGAAGAATTTCAGCC	CAACCATTATTCTGCCTCCA	65.5	133	XM_022406138
LGR5	LGR5	CTCAGCGTCTTCACCTCCT	TGGGAATGTATGTCAAAGCGT	66	130	XM_846738.2
CTNND1	p120ctn	CTCTTTCTGCCATCGCTGAC	GGGAATAGCATGTTTACCAATGAG	60	127	XM_005631199.3
PLCB4	PLCB4	CCAGTCAGGAGTATTACTAGAACG	AAAGATCCTCCTCTATATCTGTCCGAG	64.5	184	XM_854626,3
RAC1	Rac1	TCCCTTATCCTATCCGCAAA	ATGATAGGGGTGTTGGGACA	58	128	NM_001003274.2
VIM	VIMENTIN	AGCAGGAGTCAAATGAGTACC	TCCAGAGACTCATTAGTCCCT	62	84	XM_851385
**Reference genes**						
HNRNPH2	HNRPH	CTCACTATGATCCACCACG	TAGCCTCCATAACCTCCAC	61.2	151	XM_538576
RPL13	RPL13	GCCGGAAGGTTGTAGTCGT	GGAGGAAGGCCAGGTAATTC	61	87	XM_003432726
RPS5	RPS5	TCACTGGTGAGAACCCCCT	CCTGATTCACACGGCGTAG	62.5	141	XM_533568
RPS19	RPS19	CCTTCCTCAAAAAGTCTGGG	GTTCTCATCGTAGGGAGCAAG	62	95	XM_533657
SDHA	SDHA	GCCTTGGATCTCTTGATGGA	TTCTTGGCTCTTATGCGATG	61	92	DQ402985
TBP	TBP	CTATTTCTTGGTGTGCATGAGG	CCTCGGCATTCAGTCTTTTC	57	96	XM_849432
YWHAZ	YWHAZ	CGAAGTTGCTGCTGGTGA	TTGCATTTCCTTTTTGCTGA	58	96	XM_843951
**Sequencing**						
CDH3	P-Cad	TGACCATCCAGGCGACAGACAT	ATTTGGGGCACAGGACTTTGGTTG	60	804	XM_005620790.2
PLCB4	PLCB4	ATTTAATTGGCAGAAGGAAGT	ACATTGTTGGCTCGAAGTTAT	60	405	XM_854626.3

### MicroRNA assay

RNA from CMT-U27 and CIPm cells was isolated using the miRCURY™ RNA Isolation kit (Exiqon, Vedbaek, Denmark). RNA concentration was analyzed using the Nanodrop spectrophotometer ND-1000 (Thermo Scientific, Breda, The Netherlands). cDNA synthesis with 250 ng RNA was performed using the miScript II RT kit (Qiagen), according to the manufacturers protocol. A miScript miR PCR Array Dog miFinder (Qiagen) was performed on a total of 84 most abundantly expressed and best characterized microRNAs in miRBase (www.qiagen.com/nl/shop/pcr/primer-sets/miscript-miR-pcr-arrays/?catno=MIFD-001Z#geneglobe). The relative expression was measured using the miScript II RT kit on 384-well plates using the miScript SYBR Green PCR kit (Qiagen), and a standard amplification protocol according to the manufacturer. qPCR was performed on a 384 Viia (Applied Biosystems, Foster City, CA) and analyzed with QuantStudio Real-Time PCR software v1.3 (Applied Biosystems). All reactions were performed in fourfold. Data analysis for the Array was performed online (http://pcrdataanalysis.sabiosciences.com/miR), following the ΔΔCt method [66].

### DNA isolation

Genomic DNA was isolated with the DNeasy Blood and Tissue kit (Qiagen) according to the manufacturer’s protocol (cell line passage 3). DNA concentration and quality were assessed with the Qubit^®^ 2.0 fluorometer (Life Technologies, Froster City, CA, USA). Thirty nanograms of purified genomic DNA was used for the enrichment of exonic sequences and subsequent exome sequencing analysis.

### Exome sequencing

Canine mammary tumor cell line exome sequencing was performed similar to human exome sequencing protocols [67]. In brief, enrichment of exonic sequences of eight cell lines was achieved by using the SureSelect^XT^ Canine All Exon Kit (Agilent Technologies) for solid 5500 xl (Life Technologies) multiplexed sequencing of genomic DNA from each of the ten exome libraries. We obtained on average more than 134 million mappable sequencing reads per sample. Color space reads were mapped to the canFam3.1 canine reference genome with SOLiD LifeScope software version 2.1, which uses an iterative mapping approach. Single-nucleotide variants were subsequently called by the DiBayes algorithm using high-stringency calling settings, and small insertions and deletions were detected using SOLiD Small Indel Tool. All sequence variants were annotated and their effect at the DNA, RNA and protein level were predicted with an automated pipeline for canine. This pipeline was built based on the existing pipeline for the annotation of human exome sequencing projects as a template [68, 69]. In total, 8 canine mammary tumor cell lines: three with a highly active Wnt signaling and five with a low or moderate Wnt signaling [20] were exome sequenced. With canine reference genome CanFam 3.1 18000 annotated genes were mapped leading to 6653 mutated genes in total. Each individual cell line had 4000 to 5800 mutated genes. Compared the high against the low Wnt cell lines 189 genes were left over. With Panther software ((http://www.Pantherdb.org) these 189 genes were tested for a direct correlation with the Wnt signaling pathway resulted in 8 target genes with a sorting tolerant from intolerant (SIFT) score < 0.1. Further analyzing and scaling up the SIFT score was leading the key player target gene.

### siRNA

Canine sequence-specific CDH3 (GenBank: XM_848616.3) and PLCB4 (GenBank XM_014107043) siRNA was designed on the website http://rnaidesigner.termofisher.com (Thermo Scientific). Universal MOCK siRNA (ON-Target and the nontargeting pool species H (human), M (mouse) and R (rat) (Thermo Scientific) were used as negative controls for the siRNA experiments. There was no cross-silencing of nontarget genes, which was verified by BLASTing the designed siRNA sequences against the canine genome database. The sequences of the siRNA duplexes were as follows: CDH3-779 sense UUCAACAGCAACCAGCCUGUUUCCU and antisense AGGAAACAGGCUGGUUGCUGUUGAA, and PLCB4 sense UGAUAAAGCAUCCACAAUAUCUCCA and antisense UGGAGAUAUUGUGGAUGCUUUAUCA. A total of 125,000 CMT-U27 cells were transfected with 2 μl Lipofectamine 2000 (Invitrogen) transfection reagent, 50 nM siRNA and 0.8 μg DNA in 24-well Primaria plates (Corning, New York, USA). After 48 and 72 h incubation in DMEM/F12 and 10% FCS the cells were harvested for RNA isolation, protein isolation and a transcription factor (TCF)-reporter assay.

### TCF-reporter assay

Cells were seeded in a 24-well Primaria plate (Corning) at a density of 125,000 CMT-U27 cells/well and allowed to reach 80% confluency 24 h before transfection. Transfection was performed in FBS-free medium using 2 μl Lipofectamine 2000 (Invitrogen), 800 ng pTOPFLASH (TOP) or pFOPFLASH (FOP) (a gift from Prof. Dr. Hans Clevers, Hubrecht Institute, The Netherlands) and 0.5 ng human ß-actin-promoter Renilla construct [70] as an internal control. Cells were treated with the siRNA’s either or both with dasatinib (20 μM) or with GI254023X (5 μM) (Tocris, Bristol, UK). The firefly and Renilla luciferase activities were analyzed using a Dual-Luciferase Assay System (Promega, Leiden, The Netherlands) in a Centro LB 960 luminometer (Berthold Technologies, Vilvoorde, Belgium).

### Cell viability

Cell viability was analyzed by a colorimetric 3-[4,5-dimethylthiazol-2-yl] 2,5-diphenyltetrazolium bromide (MTT) assay (Sigma-Aldrich, Zwijndrecht, The Netherlands). Briefly, 15,000 CMT-U27 cells were seeded in 96-well Primaria plates (Corning) in DMEM/F12 with 10% FBS. After 24 h of incubation cell adhesion and spreading, the cells were treated with a siRNA either or both with dasatinib (Selleckchem, Munich, Germany), or with GI 254023X both dissolved in DMSO to a final stock concentration of 10 mM. Dasatinib was added in a concentration range from 1 μM to 50 μM diluted in medium to a final concentration of 0.2% DMSO and incubated for 24, 48 and 72 h (Supplementary Figure 1). 5 μM GI 254023X was added and incubated for 48 h in 0.1% DMSO. The absorbance was measured at a wavelength of 595 nm by an spectrophotometer Anthos Multimode Detector (Anthos Mikrosystem GmbH, Krefeld, Germany). IC50 curves were plotted with Sigma-plot version 12.5.

### Protein isolation and western blot

CMT-U27 cells were seeded at a density of 600,000 cells in 6-well Primaria plates (Corning) and after 24 h transient transfected with the siRNA’s. After 24, 48 and 72 h cells were washed with Hank’s balanced salt solution (Invitrogen) and lysed and scraped with RIPA buffer [20]. Twenty micrograms of protein from total cell lysate was subjected to SDS-PAGE and analyzed by Western blot. The primary antibodies used were against CDH3 (610227 antibody clone 56, AA 72-259, 1:500 dilution) (BD Biosciences, Breda, The Netherlands, SRC (Ab105215, 1:1000 dilution), pSRC (phospho S75, Ab79308, 1:500 dilution) (both from Abcam, Cambridge, UK), and β-Actin pan Ab-5 (MS-1295-P1, 1:2000 dilution) (Thermo Scientific) as a loading control. As secondary antibodies, goat anti-rabbit HRP conjugated (HAF008) and goat anti-mouse HRP conjugated (HAF007) (R&D Systems, Abingdon, UK) was used in a 1:20,000 dilution. HRP was visualized using Advance TM_enhanced chemiluminescence (ECL, GE Healthcare, Eindhoven, The Netherlands) and analyzed using GelDoc 2000 (Bio-Rad).

### Statistical analysis

Statistical analysis was conducted using IBM SPSS Statistics version 24.0.0.1 (SPSS Inc., Chicago, USA). All values were expressed as means ± SD. Statistical differences were evaluated for normal distribution, using a Student’s *t*-test for two groups (Microsoft Office, Excel version 2010) or analyzed by one-way ANOVA for multiple groups. A value of *P* < 0.05 was considered statistically significant.

## SUPPLEMENTARY MATERIALS



## Abbreviations

AA: amino acid
ADAM: a disintegrin and metalloproteinase
AKT: protein kinase B
ALDH1A1: aldehyde dehydrogenase 1 family, member A1
APC: adenomatous polyposis coli
AURKAIP1: aurora kinase A interacting protein
βACT: β-actin
BC: breast cancer
CAMK2A: calcium/calmodulin-dependent protein kinase II alpha
CDH1: E-cadherin
CDH3: P-cadherin
CMT: canine mammary tumor cell line
CSC: cancer stem cells
CSNK1D: casein kinase 1 delta
CSNK2A2: casein kinase II alpha
cSRC: rous sarcoma proto-oncogene
EC: extacellular domain
ECM: extracellular matrix
EGFR: epidermal growth factor receptor
EMT: epithelial mesenchymal transition
FAK: focal adhesion kinase
FOSL1: fos-like antigen 1
GSK3β: glycogen synthase kinase 3β
HER2/3: human epidermal growth factor receptor 2/3, ERBB2/3
IC50: half-maximum inhibitory concentration
IGV: Integrative Genomics Viewer
INO80: complex, catalytic subunit
ITGA5: fibronectin receptor alpha subunit
ITGB1: fibronectin receptor beta subunit
LEF1: lymphoid enhancer-binding factor 1
LGR5: low density-lipoprotein related protein
MET: mesenchymal-epithelial transition
miR: micro-RNA
MMP: matrix metalloprotease
mTor: mechanistic target of rapamycin
MOCK: siRNA control
nt: nucleotide
p120ctn: catenin, delta-1
PCP: planar cell polarity
PCR: polymerase chain reaction
PI3K: phosphatidylinositol-4,5-biphosphate-3-kinase
PLC: phospholipase C
PLCB4: phopholipase C, beta-4
pSRC: phosphorylated cSRC
