# Correction: P-cadherin mutations are associated with high basal Wnt activity and stemness in canine mammary tumor cell lines

**DOI:** 10.18632/oncotarget.27475

**Published:** 2020-02-04

**Authors:** Elpetra Timmermans-Sprang, Rob W.J. Collin, Arjen Henkes, Meike Philipsen, Jan A. Mol

**Affiliations:** ^1^ Department of Clinical Sciences of Companion Animals, Faculty of Veterinary Medicine, Utrecht University, Utrecht, The Netherlands; ^2^ Department of Human Genetics, Radboud University Medical Center, Nijmegen, The Netherlands; ^3^ Donders Institute for Brain, Cognition and Behaviour, Radboud University Medical Center, Nijmegen, The Netherlands

**This article has been corrected:** The name of the 2nd author has been updated as follows:

**Rob W.J. Collin**

Original article: Oncotarget. 2019; 10:2930–2946. 2930-2946. https://doi.org/10.18632/oncotarget.26873

