# Supplementary material for: P-cadherin mutations are associated with high basal Wnt activity and stemness in canine mammary tumor cell lines
Source: Oncotarget. 2019 Apr 26;10(31):2930–46. doi: 10.18632/oncotarget.26873 (PMC6508207; doi:10.18632/oncotarget.26873)
Supplement: Supplementary file 1 [file oncotarget-10-2930-s001.pdf]

P-cadherin mutations are associated with high basal Wnt activity and stemness in canine mammary tumor cell lines

SUPPLEMENTARY MATERIALS

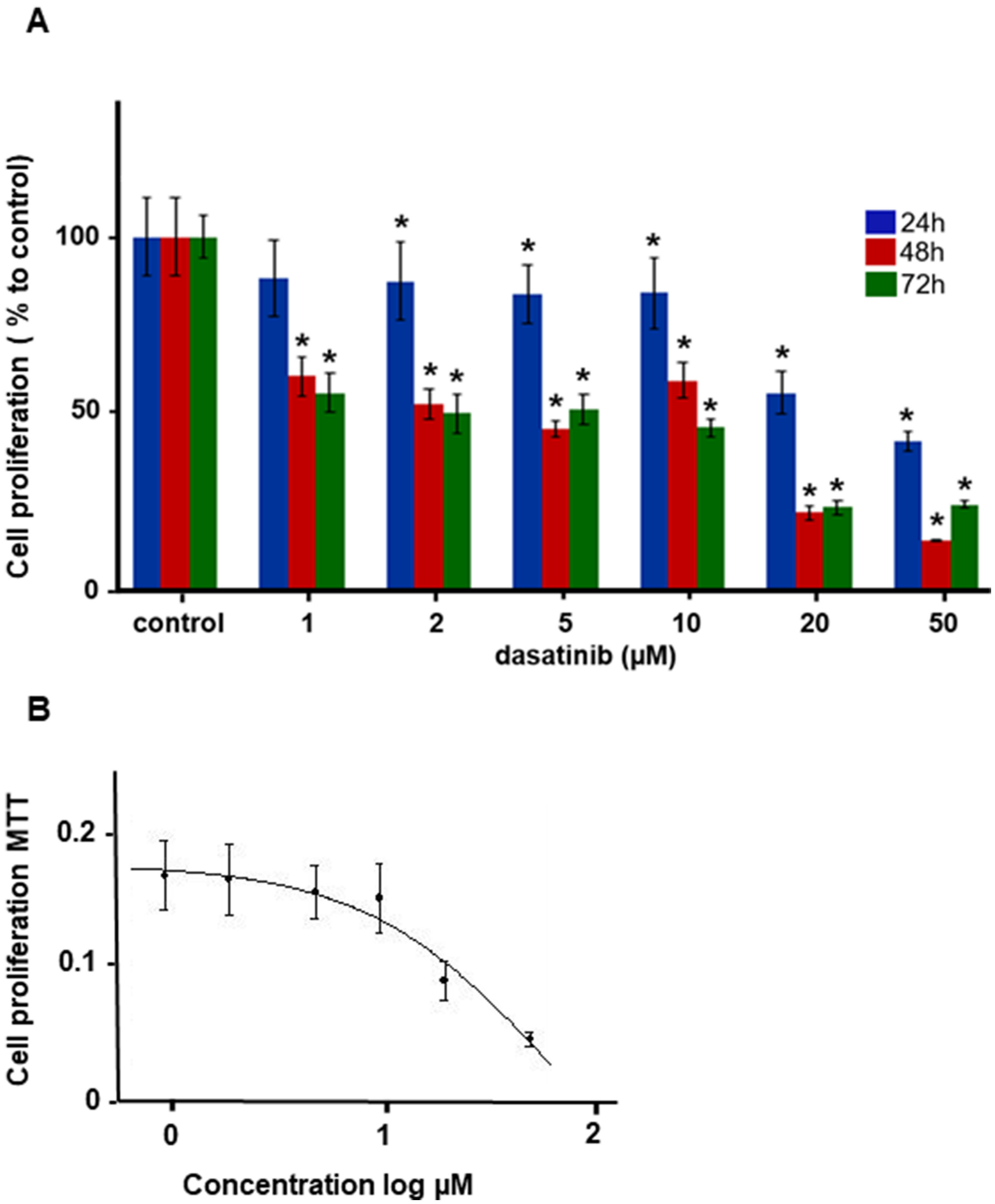

Supplementary Figure 1: The cell proliferation after treatment with dasatinib. Canine mammary tumor cells (CMT-U27) were treated with a concentration range (1–50 µM) of dasatinib. Cell proliferation was represented as percentage of 0.2% DMSO control. Statistical analysis was done on one representative experiment, out of three experiments ($n = 8$). Normal distribution was tested and *t*-test performed in Excel, $^{*}P < 0.05$ (**A**). The IC50 for dasatinib value was calculated with Sigma plot version 12.5 (**B**).

Supplementary Table 1: The assessment of canine mammary tumor cell lines used

		Cell line											
**Characteristic**		**CMT1**	**CMT–U27**	**CMT9**	**P114**	**CHMm**	**CNMm**	**CIPm**	**CIPp**				
tumor type		carcinoma	carcinoma	carcinoma	carcinoma	metastase	metastase	metastase	carcinoma				
origin		Poodle	Poodle	not known	Greyhound	Mixed breed	Maltese	Shih Tzu	Shih Tzu				
can fam number		27	28	29	30	31	32	33	34				
Wnt activity		>30	>30	>30	6	1	5	1	1				
**gene expression:**													
HER2		1,3	1,1	1	–0,2	0,6	–0,2	–1,1	–0,3				
HER3		6	5,2	5,1	–6,1	0,5	–2,6	–0,5	–0,1				
LEF1		5,6	6,8	7,8	<–6.2	<–7.1	<–6.7	<0.3	<0.1				
PTEN		–17,4	–17,6	–17,8	5,4	5,9	5,9	5,9	6,2				
ID2		1,8	4,9	5,7	–1,9	–5,5	–3,5	–0,7	–0,9				
CDH1		3,3	2	2.2	–1	3,1	–5,9	–2,7	–2,5				
CDH3		0.1	–1	0,1	2,4	2,2	–2,6	–0,7	–0,4				
ALDH1A1		6.1	4.8	4.4	–0,2	–5	<–5.0	–2,6	–1,5				
LGR5		–3,6	10,6	11,4	<–2.1	<–4.3	<–2.3	<–4.0	<–5.6				
ITGA5		–8,9	–6,5	–9,4	4.1	2.4	5.9	–1,7	–0,4				
ITGB1		–1,8	–1,2	–0,3	–0,2	1.6	0.4	–0,3	–0,3				
	**Location**									**score**	**Strand**	**Accesion number**	**Synonymous**
INO80	chr30:8102106	A	A	A	G	G	G	G	G	0,08	min	XM_844090.3	FALSE
INO80 AA		V	V	V	A	A	A	A	A				
AURKAIP1	chr5:56540446	G	G	G	A	A	A	A	A	0,1	min	XM_843641.3	FALSE
AURKAIP1 AA		P	P	P	L	L	L	L	L				
CSNK1D	chr9:99751	C	A/C	A/C	C	C	C	C	C		plus	XM_005623972.1	FALSE
CSNK1D AA		A	A/D	A/D	A	A	A	A	A				
PLCB4	chr24:12857110	T/C	T/C	T/C	C	C	C	C	C	0.55	min	XM_854626.3	FALSE
PLCB4 AA		V/I	V/I	V/I	V	V	V	V	V				
CAMK2A	chr4: 58840135	C	C	C	T	T	T	T	T		plus	XM_005619301.1	FALSE
CAMK2A AA	SA SITE												
CSNK2A2	chr2: 58429570	deletion AG	deletion AG	deletion AG							plus	XM_005617496,1	FALSE
CSNK2A2 AA	SD SITE												
RAC1	chr6: 11743627	G	G	G	C	C	C	C	C		plus	XM_005621021,1	FALSE
RAC1 AA	SA SITE												
CDH3	chr5: 80876690	G	G	G	A	A	A	A	A	1	min	XM_848616,3	FALSE
CDH3 AA 516	exon11	A	A	A	V	V	V	V	V				
CDH3	chr5: 80877348	T	T	T	C	C	C	C	C	0,36	min	XM_848616,3	FALSE
CDH3 AA 429	exon 10	I	I	I	V	V	V	V	V				
CDH3	chr5: 80879695	T	T	T	C	C	C	C	C	0,34	min	XM_848616,3	FALSE
CDH3 AA 334	exon 9	K	K	K	E	E	E	E	E				
